# Comparative transcriptome analysis reveals significant differences in gene expression between pathogens of apple Glomerella leaf spot and apple bitter rot

**DOI:** 10.1186/s12864-022-08493-w

**Published:** 2022-03-31

**Authors:** Bowen Jiang, Ting Cai, Xiaoying Yang, Yuya Dai, Kaixuan Yu, Pingping Zhang, Pingliang Li, Caixia Wang, Na Liu, Baohua Li, Sen Lian

**Affiliations:** 1 College of Plant Health and Medicine, Qingdao Agricultural University, Qingdao, 266109 China; 2 Engineering Research Center of Fruit and Vegetable Pest Precise Control of Qingdao, Qingdao, Shandong 266109 P. R. China

**Keywords:** Apple Glomerella leaf spot, Apple bitter rot, *Colletotrichum gloeosporioides* species complex, Transcriptome, Secondary metabolism, Extracellular enzymes

## Abstract

**Background:**

Apple Glomerella leaf spot (GLS) and apple bitter rot (ABR) are two devastating foliar and fruit diseases on apples. The different symptoms of GLS and ABR could be related to different transcriptome patterns. Thus, the objectives of this study were to compare the transcriptome profiles of *Colletotrichum gloeosporioides* species complex isolates GC20190701, FL180903, and FL180906, the pathogen of GLS and ABR, and to evaluate the involvement of the genes on pathogenicity.

**Results:**

A relatively large difference was discovered between the GLS-isolate GC20190701 and ABR-isolates FL180903, FL180906, and quite many differential expression genes associated with pathogenicity were revealed. The DEGs between the GLS- and ABR-isolate were significantly enriched in GO terms of secondary metabolites, however, the categories of degradation of various cell wall components did not. Many genes associated with secondary metabolism were revealed. A total of 17 Cytochrome P450s (CYP), 11 of which were up-regulated while six were down-regulated, and five up-regulated methyltransferase genes were discovered. The genes associated with the secretion of extracellular enzymes and melanin accumulation were up-regulated. Four genes associated with the degradation of the host cell wall, three genes involved in the degradation of cellulose, and one gene involved in the degradation of xylan were revealed and all up-regulated. In addition, genes involved in melanin syntheses, such as tyrosinase and glucosyltransferase, were highly up-regulated.

**Conclusions:**

The penetration ability, pathogenicity of GLS-isolate was greater than that of ABR-isolate, which might indicate that GLS-isolate originated from ABR-isolates by mutation. These results contributed to highlighting the importance to investigate such DEGs between GLS- and ABR-isolate in depth.

**Supplementary Information:**

The online version contains supplementary material available at 10.1186/s12864-022-08493-w.

## Introduction

Apple Glomerella leaf spot (GLS) and apple bitter rot (ABR) are two devastating foliar and fruit diseases of apple, which are a serious threat to apple production worldwide [1–3]. GLS is an epidemic disease leading to early defoliation and small sunken lesions (1 to 3 mm) that rarely develop into a rot on fruit [1, 4]. Apple cultivars descending from the Golden Delicious group, especially the cultivar Gala, are susceptible to GLS, and the disease may cause more than 80% defoliation and diseased fruit before harvest, reducing productivity in subsequent seasons [1, 5]. Nevertheless, apple cultivars from Red Delicious, such as the cultivar Fuji, are highly resistant to GLS [6]. GLS was first reported in the southwestern USA by Taylor in 1971 [7] when the disease was named “necrotic leaf blotch”. Later, GLS was found in Brazil but it was also given another name as “Gala leaf spot”, and finally in the 1990s, Sutton and coworkers changed the disease name to the current one as “Glomerella leaf spot” [3, 8]. In China, GLS was first reported in 2011 at Fengxian in Jiangsu province and has become a widespread disease in apple-producing areas, such as Shangdong, Hebei, Liaoning, and Gansu Provinces [1, 9]. ABR is an ordinary fruit rotting disease of apples that occurs worldwide causing extensive fruit rot [10]. About 30 to 60% of fruits rot before harvest while serious occurring in commercial orchards [11]. All apple cultivars have been considered susceptible to ABR, especially those belonging to the late-harvest group, such as Cripps Pink and Granny Smith, are particularly susceptible [12], whereas few reports on the disease infecting apple leaves.

The causal agent of GLS was identified as *Colletotrichum* species [7, 13]. More *Colletotrichum* species were described as pathogens since GLS is vigorously occurring in apple-producing regions over the years in several countries [14]. *C. fructicola* is one of the main pathogenic species causing GLS worldwide [14]. The *Colletotrichum* species have been reclassified through morphological and molecular methodologies [15–17] and organized into complexes. The GLS pathogen *C. fructicola*, *C. aenigma, C. fioriniae*, *C. alienum*, *C. siamense*, *C. tropicale* belong to the *C. gloeosporioides* species complex (CGSC) [18, 19], while *C. karstii* belong to the *C. boninense* species complex [17], and both *C. fioriniae* and *C. nymphaeae* fall within the *C. acutatum* species complex [14, 16]. The CGSC was the dominant pathogens of GLS, the complex was systematically described by Weir et al. based on phylogenetic analyses of up to eight genes in 2012, within which 22 species and one subspecies were included. All taxa accepted within this clade were morphologically similar to the broadly defined *C. gloeosporioides*, as it has been applied in the literature for around 50 years [15].

The causal agent of ABR have been identified as seven *Colletotrichum* species, including *C. fructicola*, *C. gloeosporioides*, *C. alienum*, *C. nymphaeae*, *C. siamense*, and *C. orientalis* [2, 5, 10, 20]. The *Colletotrichum* species, such as *C. fructicola*, *C. alienum*, and *C. siamense*, are common causal agents of GLS and ABR, whereas the pathogenicity of isolates that isolated from the two diseases were various on apple. For example, *C. fructicola* was identified as GLS type and ABR type according to its pathogenicity [2].

Melanized appressoria formed by *Colletotrichum* species serves as the invasive structure into the host [21]. Appressoria produce a penetration peg that penetrates the plant cuticle and cell wall layers [21]. Appressorial melanization is a key process for successful invading, and three genes *PKS1*, *SCD1*, and *THR1* are associated with the melanin biosynthesis and one regulatory gene *CMR1* plays a crucial role in the penetration [22–25]. Mutants in which the genes encoding these enzymes were disrupted were defective in appressorial melanization and their abilities to penetrate host plants [21].

Pathogens of ABR or GLS exhibit a particular organ specialization being able to cause quite different symptoms [2, 11, 26]. However, the mechanisms for such differences are unclear until now although several reports tried to make clear the relations between GLS- and ABR-isolates. The genotypes causing GLS were may be originated from ABR-isolates under the selection force of susceptible apple Gala [27]. The difference in the ability to infect fruits and leaves could be because of extracellular enzymes produced by the fungi, which degrade the plant cuticle and cell wall components while invading [26]. The extracellular enzymes produced by ABR-isolate, such as lipolytic enzymes, proteolytic enzymes, pectin lyase (PNL), polygalacturonase (PG), and laccase (LAC) had higher activity than that produced by GLS-isolate in vitro [26]. However, those differences in extracellular enzymes of ABR- and GLS-isolate have not been observed in vivo [26].

In this context, the transcriptome profile comparison of GLS- and ABR-isolates were conducted through RNA-Seq. GLS-isolate GC20190701 and ABR-isolates FL180903, FL180906 were showed a basal gene expression pattern. The transcriptional variations among the three isolates were further identified. In addition, the potential roles for the identified DEGs between GLS- and ABR-isolates were analyzed. These results will contribute to a better understanding of the mechanism of difference between GLS- and ABR-isolates and provide insight into the relations of GLS- and ABR-isolates.

## Results

### The pathogenicity of GLS- and ABR-isolates GC20190701, FL180903, FL180906 were diverse to gala apple

**Fig. 1:**
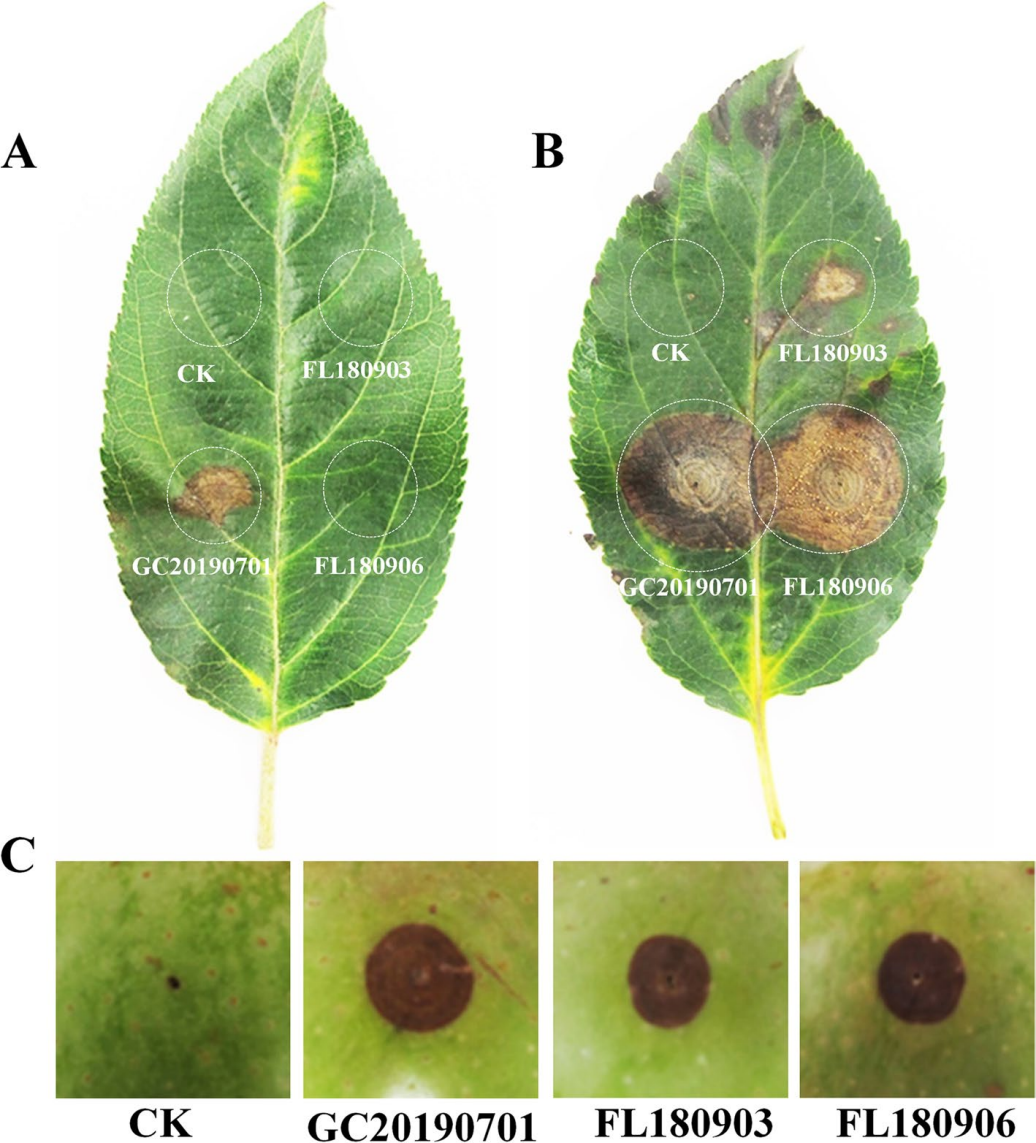
Lesions caused by the *Colletotrichum aenigma* isolate GC20190701 and *C. gloeosporioides* isolates FL180903, FL180906 on leaves and fruits of Gala apple inoculated with conidia at 10 days after inoculation. **A** GC20190701 infected Gala leaves causing necrotic spots, ABR-isolates FL180903 and FL180906 could not infect on Gala leaves by the conidia. **B** All the three isolates infected Gala apple leaves by the conidia through micro-wound. **C** The three isolates infected Gala apple fruit through micro-wound by conidia, and cause rot lesions

The GC20190701 was isolated from leaves of Gala apple, which showed a typical symptom of apple Glomerella leaf spot, and the FL180903, FL180906 were isolated from Fuji apple fruits that showed a typical symptom of apple bitter rot. The pathogenicity of the three isolates was diverse on apple leaves and fruits. The conidia of GC20190701 could infect Gala leaves, and cause necrotic spots, but the conidia of isolates FL180903 and FL180906 could not complete the infection on Gala leaves without a wound (Fig. 1A). Whereas, the three isolates could infect Gala apple leaves by the conidia through micro-wound (Fig. 1B), the necrotic spot caused by GC20190701 and FL180906 were similar in size, which was larger significantly than that caused by FL180903. The three isolates could infect apple fruit through micro-wound by conidia, and cause rot lesions (Fig. 1C). The rot lesions caused by GC20190701 were larger than those caused by FL180903 or FL180906, while rot lesions were similar in size caused by FL180903 and FL180906.

### Multi-locus phylogenetic analysis of GLS- and ABR-isolates GC20190701, FL180903, FL180906

**Fig. 2:**
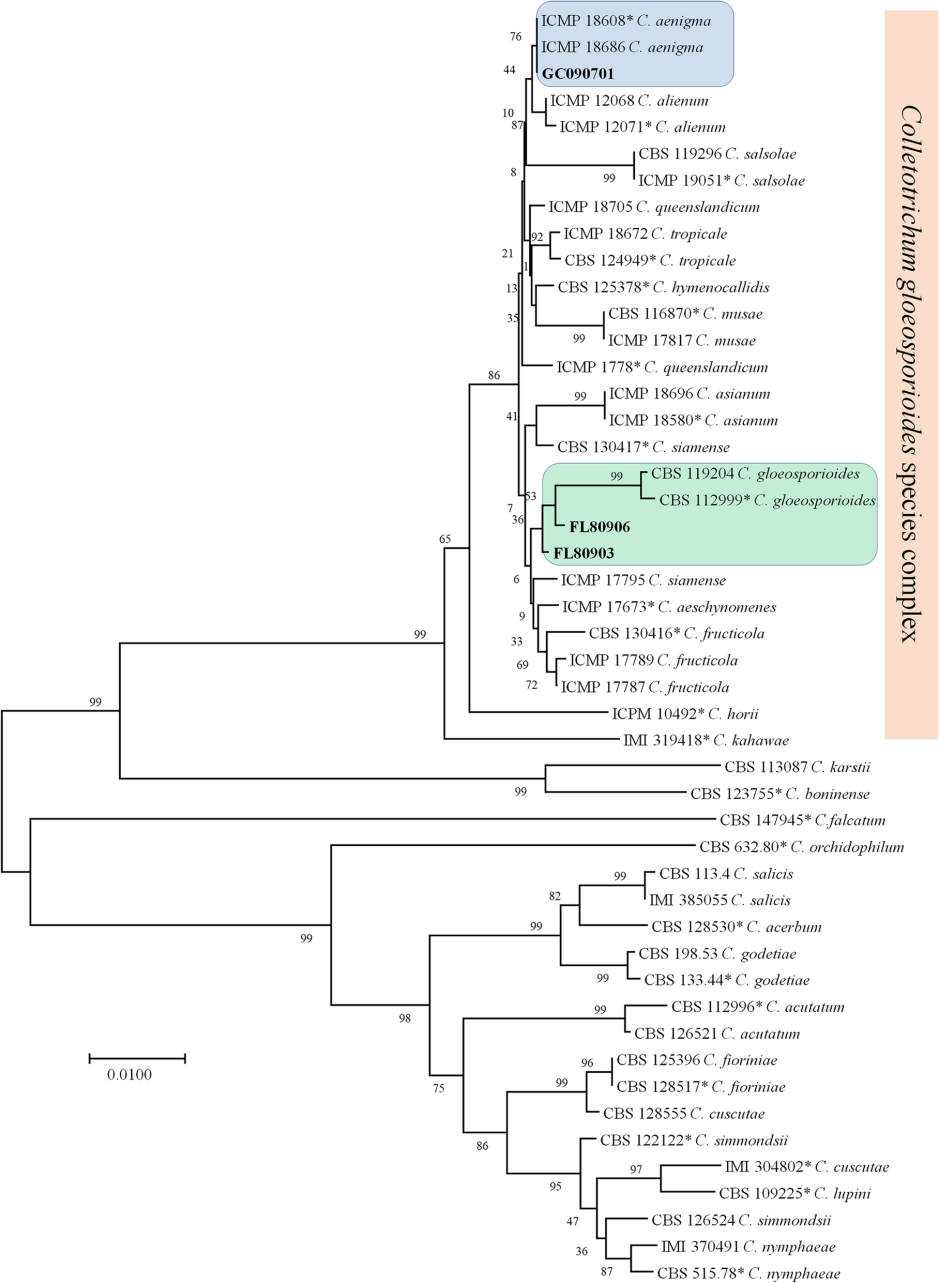
Phylogenetic trees of the *Colletotrichum aenigma* isolate GC20190701 and *C. gloeosporioides* isolates FL180903, FL180906. The phylogenetic tree was constructed using the Kimura 2-parameter method with bootstrap resampling (1000 neighbor-joining replications) and concatenated ITS, GAPDH, TUB2, ACT, CHS-1 sequences. The percentage of replicate trees in which the associated taxa clustered together in the bootstrap test (1000 replicates) is shown next to the branches. The isolates used in this study are shown in bold. Different background colors indicate different species of *Colletotrichum* strains. Ex-type and authentic cultures are marked by an asterisk (*). Evolutionary analyses were conducted in MEGA7.0.14

To elucidate the phylogenetic position of the GLS- and ABR-isolates GC20190701, FL180903, FL180906, five genes, such as *ITS*, *GAPDH*, *TUB2*, *ACT*, *CHS-1*, were concatenated to form a supermatrix of 1966 bp, and the phylogenetic analysis of the concatenated data set was conducted using the neighbor-joining (NJ) method. The results showed that GC20190701 were clustered in the same clade with *C. aenigma*, however, FL180903 and FL180906 were clustered in the same clade with *C. gloeosporioides* (Fig. 2). The three isolates GC20190701, FL180903, and FL180906 were closely related species, which were clustered in *Colletotrichum gloeosporioides* species complex (Fig. 2).

### Transcriptome analysis of GLS- and ABR-isolates GC20190701, FL180903, FL180906

To comprehend the mechanism of pathogenicity of the three *Colletotrichum* isolates GC20190701, FL180903, and FL180906, transcriptome comparison of three *Colletotrichum* isolates were performed. The mycelia were harvested from PDA medium at 96 hpi, and RNA was extracted and purified for transcriptome sequence. A total of nine biological samples, three biological repeats for each isolate were sequenced using next-generation sequencing on the Illumina sequence platform. Totally 56.58 billion bp clean data was achieved, which were further spliced to 38,844 assembled unigenes (Table 1, Table S1). The total length of unigenes was 51,182,491 bp, the average length was 1318 bp, and the longest one was 23,113 bp (Table 1).

**Table 1:** Summary of assembled sequences in the *Colletotrichum aenigma* isolate GC20190701 and *C. gloeosporioides* isolates FL180903, FL180906

Index	Contig	Transcript	Unigene
Total Length (bp)	70,412,489	204,569,566	51,182,491
Sequence Number	182,409	96,684	38,844
Max. Length (bp)	28,478	23,113	23,113
Mean Length (bp)	386.0	2115.9	1317.6
N50 (bp)	1670	3718	3061
N50 Sequence No.	9219	18,204	5019
N90 (bp)	134	1176	439
N90 Sequence No.	120,590	53,845	20,756
GC%	52.7	54.9	53.8

### Functional annotation and classification of unigenes

Gene annotation was performed to analyze the functions of the expressed genes. Totally 24,783 (63.80% of 38,844) unigenes were annotated in at least one database. A total of 23,089 unigenes (59.44% of 38,844) were annotated in NCBI non-redundant protein sequences (NR), and 14,445 unigenes (37.19%), 7662 unigenes (19.73%), 10,049 unigenes (19.73%), 19,696 unigenes (50.71%), 15,372 unigenes (39.57%), were annotated in GO, KEGG, Pfam, eggNOG, and Swiss-Prot, respectively (Table S2). There were 3655 unigenes (9.41%) were annotated in all the databases (Table S2).

**Fig. 3:**
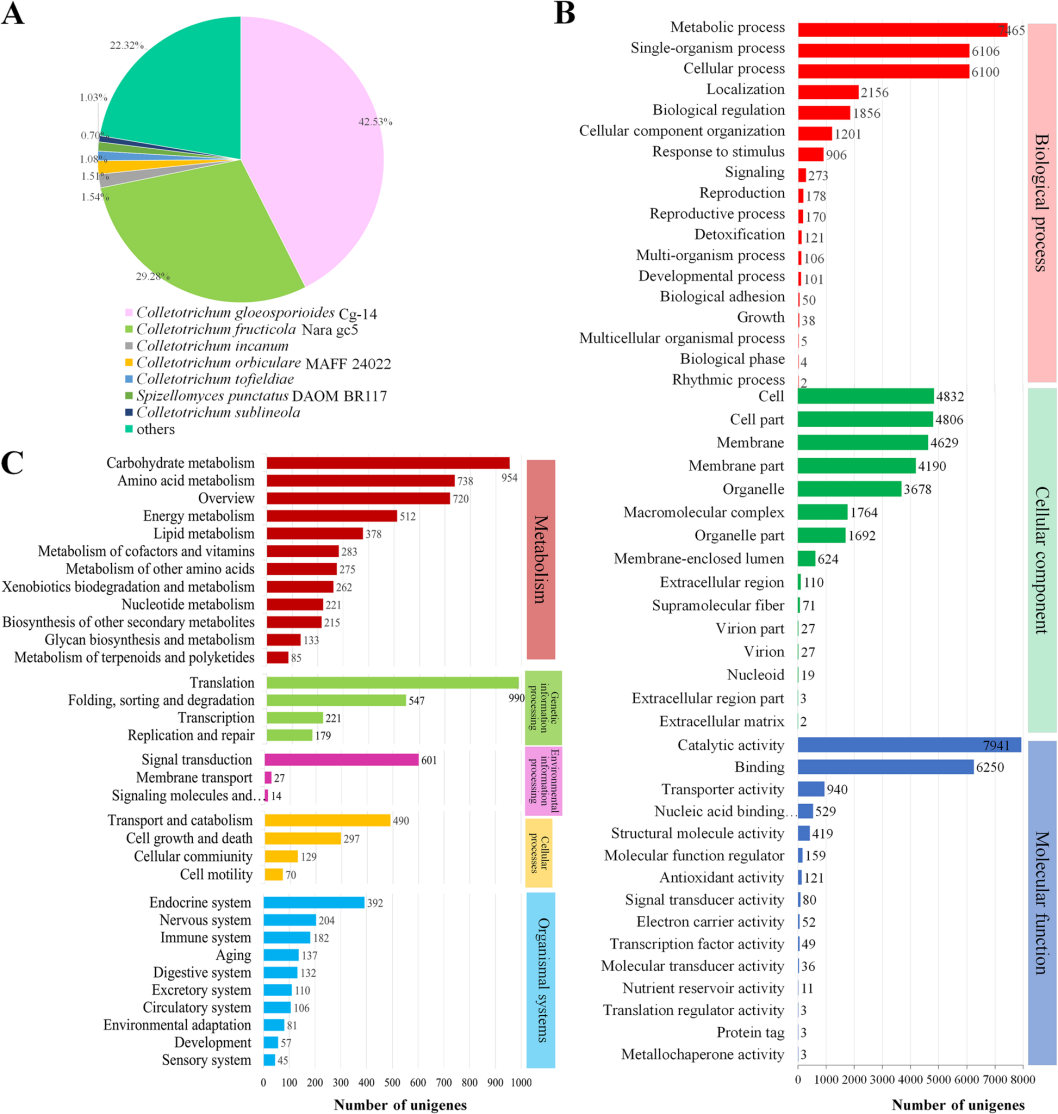
Functional annotation and classification of unigenes of the *Colletotrichum aenigma* isolate GC20190701 and *C. gloeosporioides* isolates FL180903, FL180906. **A** The best matched to the known nucleotide sequences were *C. gloeosporioides* CG-14 (42.53%) and *C. fructicola* Nara gc5 (29.28%). **B** The abundant GO terms of the biological process categories (indicated by red), cellular component categories (indicated by green), and molecular function (indicated by blue). **C **The 7662 annotated unigenes were assigned to 33 KEGG pathways. Metabolism pathways (48.8%, indicated by red), Genetic information processing (19.8%, indicated by pea green), Environmental information processing (6.6%, indicated by purple), Cellular processes (10.1%, indicated by yellow), and Organismal systems (14.8%, indicated by light blue)

The annotated unigenes were compared to known nucleotide sequences of microbe species. The best matched to the known nucleotide sequences were *C. gloeosporioides* CG-14 (42.53%) and *C. fructicola* Nara gc5 (29.28%). Only 5.86% of unigenes matched to other four *Colletotrichum* species (Fig. 3A).

Clusters of Gene Ontology (GO) classification were calculated by BLAST2GO. A total of 14,445 annotated unigenes (37.19% of 38,844) were assigned to at least one of the 48 GO terms (Fig. 3B, Table S3). The unigenes were assigned to biological process, cellular component, and molecular function, respectively. The unigenes in the molecular function category were abundant in catalytic activity (GO:0003824) and binding functions (GO:0005488) (Fig. 3B). The biological process category of the unigenes was predominantly associated with metabolic process (GO:0008152), single-organism process (GO:0044699), and cellular process (GO:0009987) (Fig. 3B). The cellular component category of the unigenes was predominantly associated with cell (GO:0005623), cell part (GO:0044464), and membrane (GO:0016020) (Fig. 3B). The fundamental biological processes of the isolates GC20190701, FL180903, FL180906 were identified according to the above findings.

Biological functions of annotated unigenes in different pathways were systematically evaluated using the Kyoto Encyclopedia of Genes and Genomes (KEGG) pathway database. A total of 7662 annotated unigenes were matched to the KEGG database, which were assigned to 33 KEGG pathways (Fig. 3C, Table S4). Metabolism pathways (4776 unigenes, 48.8%), genetic information processing (1937 unigenes, 19.8%), and organismal systems pathways (1446 unigenes, 14.8%) were the three dominant categories. The subcategory translation pathway in genetic information processing (990 unigenes), carbohydrate metabolism (954 unigenes), and amino acid metabolism (738 unigenes) in metabolism pathways were the three dominant subcategories (Fig. 3C, Table S4).

### Differential expression analysis of unigenes of isolates GC20190701, FL180903, FL180906

**Fig. 4:**
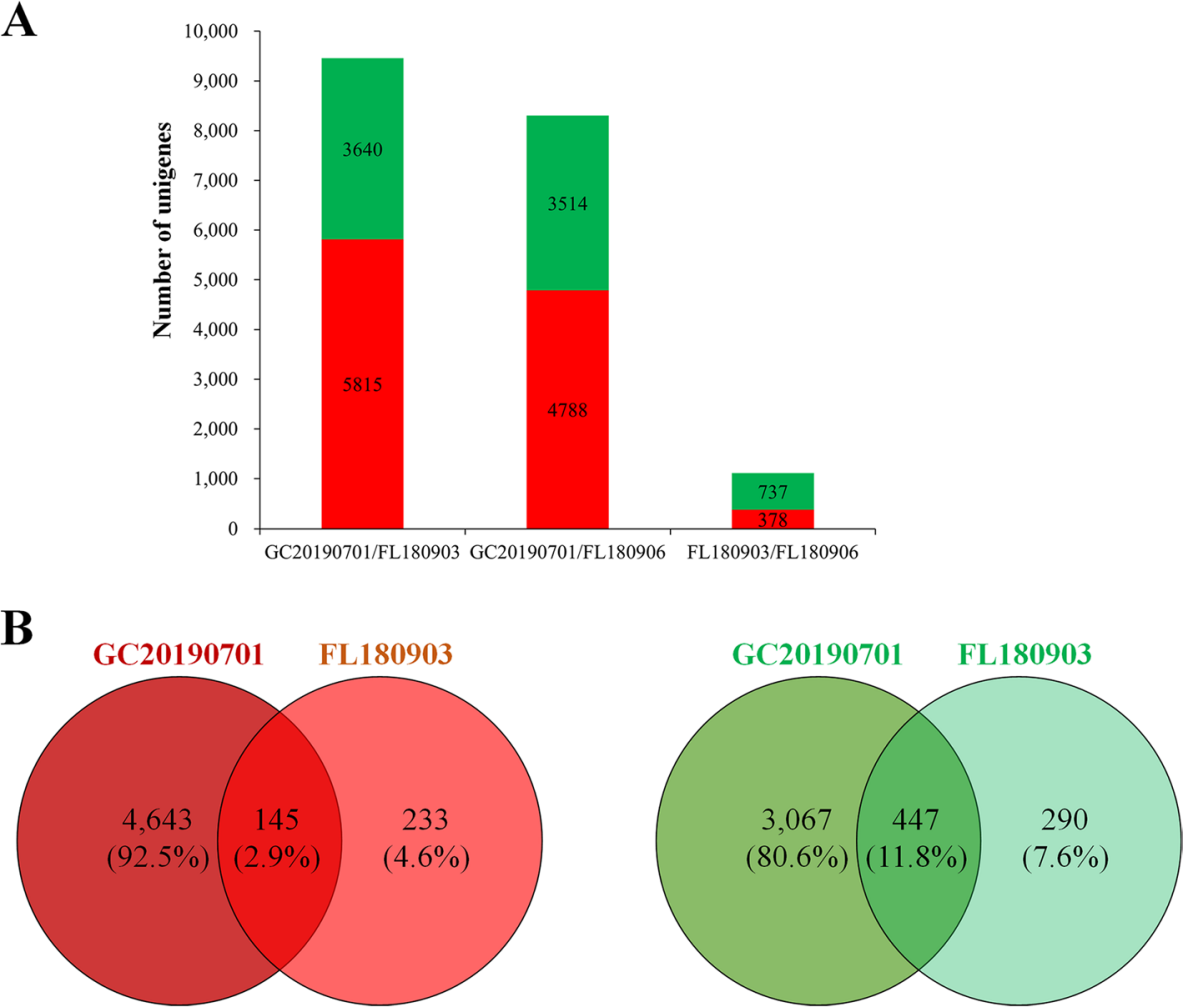
The number of differentially expressed genes (DEGs) among the *Colletotrichum aenigma* isolate GC20190701 and *C. gloeosporioides* isolates FL180903 and FL180906. **A** The DEGs were compared in pairs of the three isolates. In the isolate GC20190701, a total of 8302 unigenes were differentially expressed compared to the isolate FL180906, and there were 9455 DEGs between the isolate GC20190701 and FL180903. Whereas only a total of 1115 DEGs between the isolate FL180906 and FL180903. **B** Venn diagram displaying the distribution of DEGs (genes with > 2-fold change in expression) in GC20190701 and FL180903 compared with FL180906

To analyze the differential expression of unigenes (DEGs) among the three isolates, the transcriptomes were compared in pairs, and the expression level of unigenes was calculated by the FPKM method. In the isolate GC20190701, a total of 8302 unigenes were differentially expressed compared to the isolate FL180906, and the up-regulated unigenes and down-regulated unigenes were 4788 and 3514, respectively (Fig. 4A). There were 9455 DEGs between the isolate GC20190701 and FL180903, and the up-regulated genes and down-regulated genes were 5185, and 3640 compared to FL180903 (Fig. 4A). Whereas only a total of 1115 DEGs between the isolate FL180906 and FL180903, the up-regulated unigenes were 378, and the down-regulated unigenes were 737 compared with FL180906 (Fig. 4A).

A total of 145 up-regulated unigenes and 447 down-regulated unigenes overlapped between GC20190701 and FL180903 compared with FL180906 (Fig. 4B). The transcriptome expression profile of GC20190701 was quite different from that isolates FL180903 and FL180906, whereas the transcriptome expression profile of FL180903 was not that different from that FL180906.

### Gene ontology and KEGG enrichment analysis of DEGs of isolates GC20190701, FL180903, FL180906

**Fig. 5:**
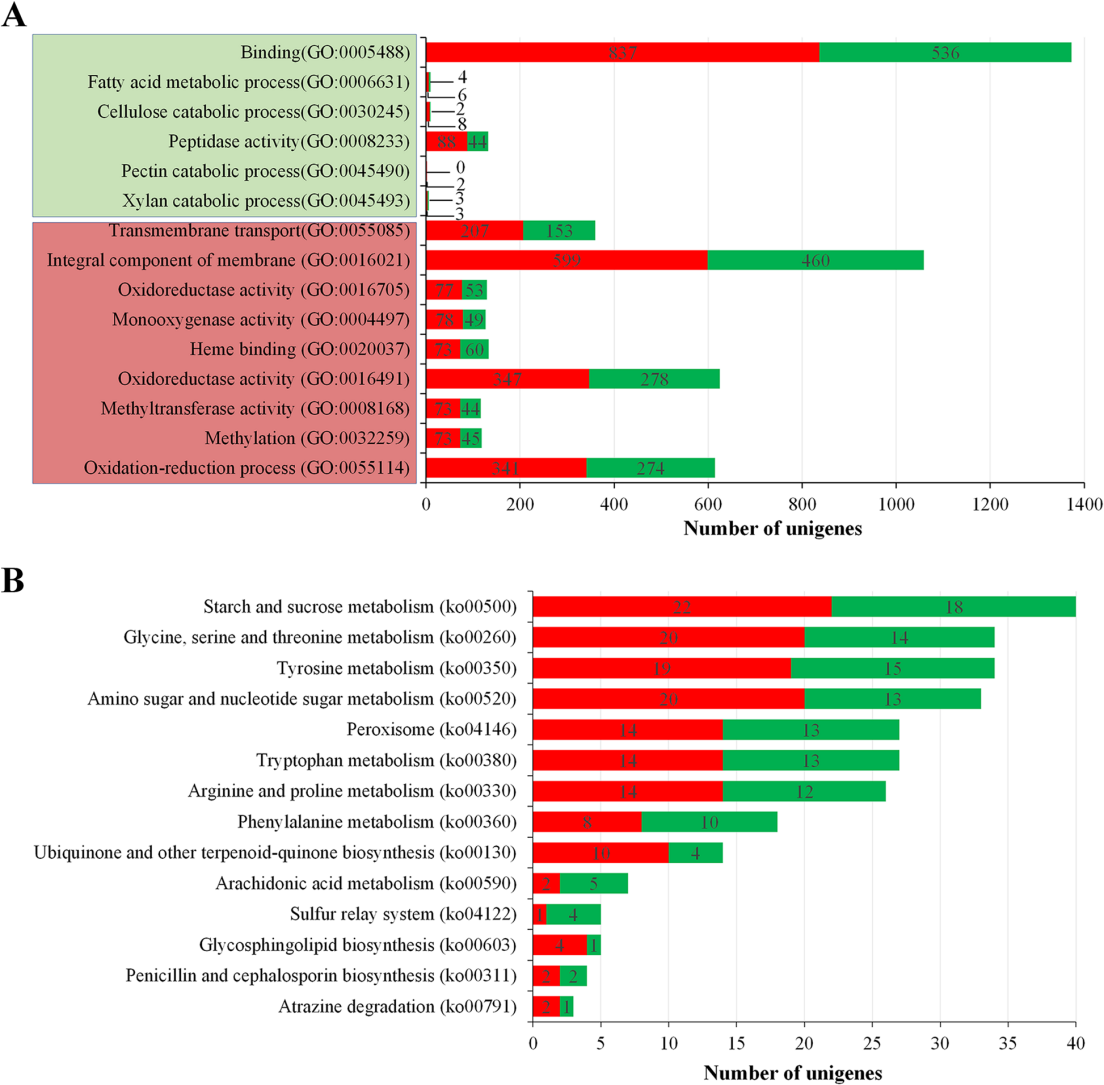
The number of Go terms and KEGG pathways by Gene ontology and KEGG enrichment analysis of DEGs between the *Colletotrichum aenigma* isolate GC20190701 and *C. gloeosporioides* isolate FL180906. **A** Enriched GO categories involved in the biosynthesis of secondary metabolites (light green) and categories of degradation of various cell wall components (light red). Bars indicate the gene numbers involved in the Go terms, and green and red bars indicate groups of down- and up-regulated genes, respectively. **B** Enriched KEGG pathways of the DEGs between GC20190701 and FL180906. A total of 14 pathways were significantly enriched with *P* values < =0.05. Bars indicate the gene numbers involved in the pathways, and green and red bars indicated the down- and up-regulated genes, respectively

Gene ontology (GO) enrichment analyses were conducted using a cut-off of *P* < 0.05 to determine the functional roles of DEGs of the three isolates. The DEGs of isolate GC20190701 and FL180906 were assigned in 3565 enriched GO terms. There were 184 significant enriched GO terms (*p* < 0.05), which the numbers related to biological process, molecular function and cellar component were 89, 81, and 14, respectively (Table S5). The significantly enriched GO categories of DEGs of isolate GC20190701 and FL180906 included those involved in the biosynthesis of secondary metabolites, including oxidation-reduction process (GO:0055114), methylation (GO:0032259), methyltransferase activity (GO:0008168), oxidoreductase activity (GO:0016491), heme binding (GO:0020037), monooxygenase activity (GO:0004497), oxidoreductase activity (GO:0016705), transmembrane transport (GO:0055085), and integral component of membrane (GO:0016021) (Fig. 5A, Table S5). However, the categories of degradation of various cell wall components (including catabolism of cellulose (GO:0030245), xylan (GO:0045493), pectin (GO:0045490)), peptidase activity (GO:0008233), fatty acid metabolic processes (GO:0006631), and binding (GO:0005488) activities were not significantly enriched (*P* > 0.05) (Fig. 5A, Table S5).

The DEGs of isolate FL180903 and FL180906 were assigned in 909 enriched GO terms. Totally 109 significant enriched GO terms (*p* < 0.05) were identified, the numbers related to biological process, molecular function, and cellar component were 43, 61, and 5, respectively (Table S6).

KEGG enrichment analyses were performed to identify the basal level biological pathways of the three isolates. All DEGs between GC20190701 and FL180906 were enriched into 110 KEGG pathways (Table S7). A total of 14 pathways were significantly enriched with *P* values < =0.05 (Fig. 5B, Table S7). The DEGs between FL180903 and FL180906 were enriched into 60 KEGG pathways, and eight of which were significantly enriched (Table S8).

### Functional analysis of the genes that differentially expressed in GLS-pathogen and ABR-pathogen

To compare the function of DEGs of GLS- and ABR-pathogen, we first removed the DEGs from GC20190701 which were co-differential expressed with FL180903 compared with the expression of FL180906 since the FL180903 and FL180906 were common pathogens of ABR. Second, we selected the highly differential expression genes with a |log2FoldChange| > 3, the *p* value< 0.01, and the adjusted *p*-value < 0.01 from the transcriptome of GC20190701. A total of 1124 DEGs were selected, and 649 genes were up-regulated, 475 genes were down-regulated compared with the expression of FL180906 (Table S9).

The functions of the 1124 DEGs were analyzed by searching the Swissprot database. Among the DEGs, 42 unigenes associated with the biosynthesis of secondary metabolites including cytotoxin, mycotoxin, and antibiotics were revealed, of which 30 unigenes were up-regulated whereas 12 were down-regulated (Table 2). There were 17 cytochrome P450, five methyltransferase protein, three short-chain dehydrogenase reductases, three FAD binding proteins, two multicopper oxidases, two efflux pump antibiotic resistance, two NmrA-like family proteins were involved in the 42 DEGs, besides that one of each Mgt family protein, ABC-2 type transporter, 4-hydroxyacetophenone monooxygenase, Aminotransferase classes I and II family protein, dynamin GTPase, integral membrane protein, oxidoreductase protein, thioredoxin were also included in the 42 DEGs (Table 2).

**Table 2:** The selected highly differential expression genes in GLS-isolates compared with ABR-isolate associated with the biosynthesis of secondary metabolites and pathogenicity

Gene_id	log2^**FoldChange**^	***p***-value	***p*** adj	Length	NR description
DN11786_c0_g1	10.1726	1.87481E-17	1.35521E-15	2229	Cytochrome P450
DN13770_c0_g1	7.7964	7.73587E-12	2.28663E-10	1861	Cytochrome P450
DN20588_c0_g1	6.4786	6.2621E-08	9.9134E-07	2105	Cytochrome P450
DN32057_c0_g1	5.7993	6.67483E-12	1.98619E-10	2533	Cytochrome p450 pisatin
DN29395_c0_g1	5.2268	1.57697E-10	3.86549E-09	1981	Cytochrome p450 family protein
DN28542_c0_g1	5.0673	1.34696E-09	2.79951E-08	1931	Cytochrome P450
DN35922_c0_g1	4.5778	7.70861E-09	1.41189E-07	5435	Cytochrome P450
DN28908_c0_g1	3.9370	0.001122151	0.007588586	2120	Cytochrome p450 oxidoreductase
DN30184_c0_g1	3.7122	1.71709E-06	2.10697E-05	2691	Cytochrome p450 family protein
DN33241_c0_g1	3.6714	1.62331E-06	2.00374E-05	2984	Benzoate 4-monooxygenase cytochrome p450
DN28885_c0_g1	3.0654	0.000521922	0.003845902	1748	Cytochrome P450
DN28563_c0_g1	−3.3410	0.000871754	0.006063741	2597	Cytochrome P450
DN33475_c0_g1	−3.4703	4.16395E-06	4.78347E-05	5798	Cytochrome P450
DN32532_c1_g1	−3.8683	0.000716447	0.005092608	2717	Cytochrome P450
DN28222_c0_g1	−5.0447	0.000139771	0.00117946	2290	Cytochrome P450
DN28049_c0_g2	−5.4077	6.91285E-09	1.27362E-07	2119	Cytochrome P450
DN33453_c0_g1	−5.6123	8.05501E-11	2.05531E-09	2624	Cytochrome P450
DN31973_c0_g3	3.7649	5.08745E-06	5.77806E-05	1320	Methyltransferase domain-containing protein
DN31973_c0_g2	8.8234	4.8846E-17	3.32997E-15	4825	Methyltransferase domain-containing protein
DN31000_c2_g2	5.6164	1.58532E-10	3.88292E-09	1677	Methyltransferase domain-containing protein
DN30174_c1_g1	4.2519	7.75456E-07	1.01852E-05	1595	O-methyltransferase
DN31843_c0_g1	5.6821	1.30938E-05	0.000136617	1218	SAM dependent methyltransferase, putative
DN34847_c0_g2	3.1430	0.000442733	0.003313322	1568	Short-chain dehydrogenase reductase
DN28087_c0_g1	4.8386	6.61206E-06	7.34398E-05	1359	Short-chain dehydrogenase reductase
DN28633_c0_g2	6.3343	3.4573E-09	6.71105E-08	2386	Short-chain dehydrogenase reductase family
DN31745_c1_g2	−8.6067	6.59755E-07	8.80663E-06	4113	FAD binding domain-containing protein
DN29628_c0_g1	3.8020	3.77962E-06	4.37747E-05	2911	FAD binding domain-containing protein
DN27472_c0_g1	3.2978	0.000250727	0.001990167	1652	FAD binding domain-containing protein
DN36036_c8_g4	4.4081	1.53968E-08	2.69184E-07	416	Multicopper oxidase
DN36036_c7_g1	6.9116	1.84547E-09	3.75298E-08	212	Multicopper oxidase
DN35008_c0_g1	−4.9712	0.000773001	0.005452308	1069	Efflux pump antibiotic resistance
DN36131_c2_g2	8.3251	1.12354E-18	1.00586E-16	2959	Efflux pump antibiotic resistance
DN24662_c0_g1	−11.5043	2.41435E-05	0.000239094	1055	NmrA-like family protein
DN23599_c0_g1	−4.2513	1.39353E-05	0.000144477	1143	NmrA-like family protein
DN22384_c0_g1	4.1657	0.001295912	0.00860974	1331	4-hydroxyacetophenone monooxygenase
DN28500_c0_g1	5.7415	3.74839E-09	7.23103E-08	1430	Aminotransferase classes I and II family protein
DN29598_c0_g1	7.9391	5.26601E-15	2.51244E-13	4129	Dynamin GTPase
DN35303_c1_g1	11.6073	4.60773E-24	1.49535E-21	1116	Integral membrane protein
DN28459_c0_g3	3.4975	0.000907408	0.006279499	533	Oxidoreductase family protein
DN30510_c0_g5	10.9541	2.24851E-21	3.70648E-19	533	Thioredoxin
DN20622_c0_g1	−5.6331	3.54404E-06	4.12456E-05	1468	MGT family
DN32373_c0_g2	−3.8836	5.84537E-06	6.56498E-05	4913	ABC-2 type transporter
DN36403_c6_g2	4.2504	6.06648E-08	9.65771E-07	4007	CFEM domain-containing protein
DN21054_c0_g1	4.4493	0.000368273	0.002816287	1726	CFEM domain-containing protein
DN27480_c0_g1	−13.0590	1.68911E-08	2.93171E-07	4310	CFEM domain-containing protein
DN26894_c0_g1	4.4476	5.68732E-06	6.399E-05	1644	Rhamnogalacturonate lyase
DN21088_c0_g1	3.7560	1.04166E-05	0.000110647	1791	Beta-glucosidase
DN33240_c0_g2	6.2602	1.4174E-06	1.77203E-05	2190	Tyrosinase 2
DN34063_c0_g2	6.3368	7.18132E-12	2.1308E-10	3030	Tyrosinase precursor
DN30086_c0_g1	3.3697	2.0996E-05	0.000210196	1749	PHB depolymerase family esterase
DN32627_c0_g1	3.2690	1.9454E-05	0.000195765	3296	Endoglucanase III
DN36348_c7_g7	6.0139	6.48164E-07	8.6742E-06	578	Serine carboxypeptidase
DN34219_c0_g1	3.3009	1.30281E-05	0.000136023	2526	Carboxypeptidase s1
DN28578_c1_g1	5.6735	2.13051E-08	3.62909E-07	1939	Carboxypeptidase 2

Four genes associated with the degradation of the host cell wall were identified, which were up-regulated. One Rhamnogalacturonate lyase (DN26894_c0_g1), two glucosidases (DN21088_c0_g1, DN32627_c0_g1), involve in degradation of cellulose, and one acetylxylan esterase (DN30086_c0_g1) involves in degradation of xylan.

Two tyrosinase (DN33240_c0_g2, DN34063_c0_g2), and one glucosyltransferase (DN25823_c0_g1) were up-regulated, which were involved in melanin synthesis. Three CFEM domain-containing proteins, which were associated with pathogenicity were also identified. Two of them (DN36403_c6_g2, DN21054_c0_g1) were up-regulated, and one (DN27480_c0_g1) were down-regulated. Three carboxypeptidases (DN36348_c7_g7, DN34219_c0_g1, DN28578_c1_g1) were identified, which were associated with pathogenicity, and both of them were up-regulated.

### Validation of RNA-Seq data by quantitative real-time RT-PCR

**Fig. 6:**
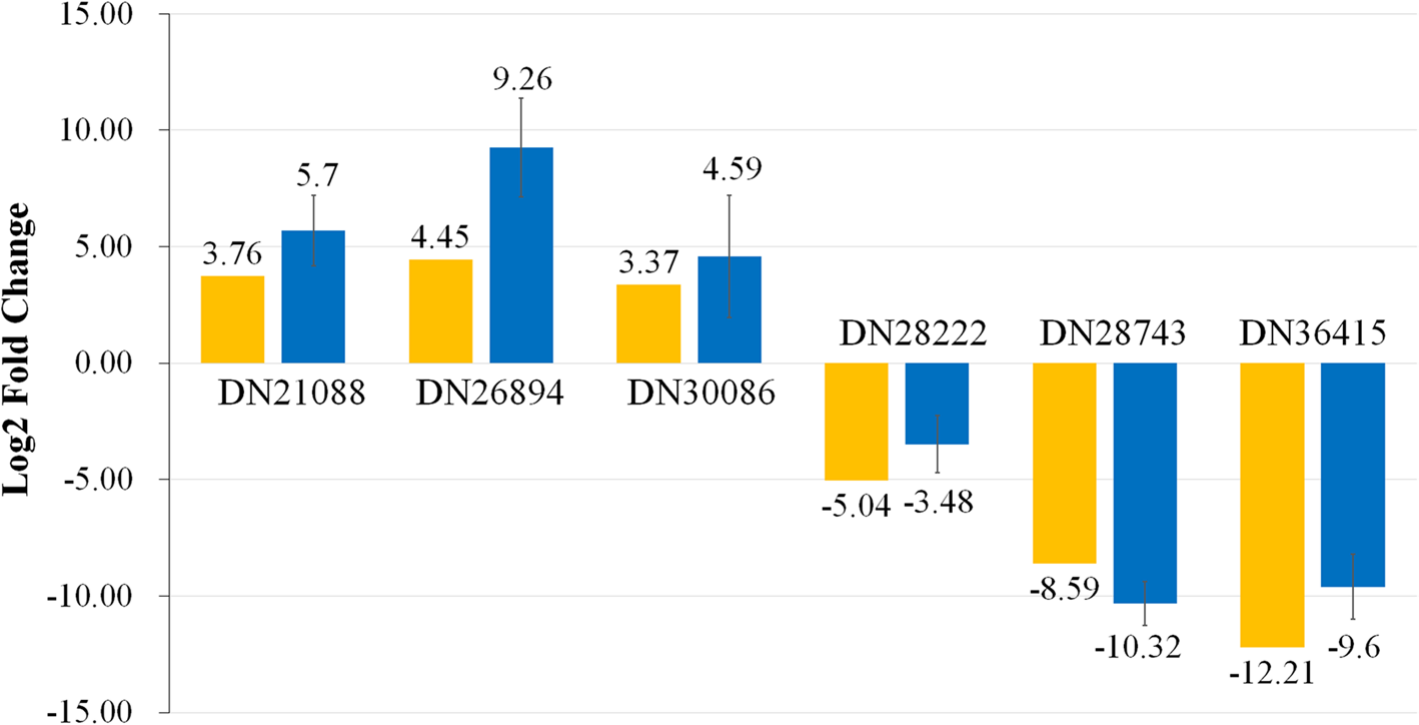
Quantitative Real-Time PCR (qRT-PCR) validation of selected unigenes. The relative expression level of each selected gene was determined by the 2^−ΔΔCT^ method. The yellow bars represent the data of RNA seq, and the blue bars represent the data of qRT-PCR. The experiments were conducted in triplicates

We validated the RNA-Seq data by quantitative RT-PCR for six representative genes that showed strong up-regulation or down-regulation in GC2190701 compared with FL180906. The genes used for validation, their log2 fold change, and the primer sequences are presented in Table S10. For quantitative RT-PCR, we prepared new samples following the same procedures that were used to prepare samples for RNA-Seq. The expression patterns of the selected six genes all agreed with the RNA-Seq results (Fig. 6), suggesting that the RNA-Seq results were reliable in this study.

## Discussion

Apple Glomerella leaf spot (GLS) and apple bitter rot (ABR) are two serious plant diseases that threaten the production of apples worldwide. The causal agents of GLS and ABR both belong to the genus *Colletotrichum*, and there are several common species causing both GLS and ABR. However, the common causal agents exhibit a particular organ specialization, which causes quite different symptoms on apples. Although several reports tried to explore the mechanism for such difference [2, 26, 27], the causes for such differences remain unknown until now. In context, we performed a transcriptome comparison of three GLS- and ABR-isolates, GC20190701, FL180903, and FL180903. A relatively large difference was discovered between the GLS- and ABR-isolate and quite many differences in expression genes associated with pathogenicity were revealed.

Using the multi-locus phylogenetic analysis, we made it clear that the isolate GC20190701 belonged to *C. aenigma*, whereas FL180903 and FL180906 belonged to *C. gloeosporioides*, however, the three isolates were all fall in *C. gloeosporioides* species complex (CGSC), and taxa accepted within this clade were morphologically similar each other [15]. We acquired high-quality transcriptome data by sequencing nine samples, and the transcripts were assigned to the genus *Colletotrichum* (Fig. 2A), therefore, the transcriptome data meet the requirement for further comparing analysis.

The transcriptome expression patterns were divergent in the three isolates GC20190701, FL180903, and FL180906. A total of 1115 unigenes were differentially expressed between FL180903 and FL180906, both of which cause ABR on fruit (Fig. 4A). Nevertheless, the number of DEGs was 9455 and 8302 in GC20190701 compared with FL180903 and FL180906, respectively (Fig. 4A). As a causal agent of GLS and ABR, the isolates not only cause the various symptoms but also possess significant differences in the transcriptome profiles.

The enrichment of GO terms and KEGG analysis of DEGs were conducted. The DEGs between the GC20190701 and FL180906 were significantly enriched in secondary metabolites, however, the categories of degradation of various cell wall components did not significantly enrich. The secondary metabolites were significantly divergent between the GLS-isolate and ABR-isolate, while the degradation enzyme may not change that much. However, the enrichment of GO terms of DEGs between isolate FL180903 and FL180906 had identical trends with that DEGs between GC20190701 and FL180906 although the number of unigenes involved in the GO terms was less than that of GC20190701 and FL180906.

We combined several measures in the analysis procedure to reveal rigorous DEGs between GLS-isolate and ABR-isolate. First, we removed the DEGs common in GC20190701 and FL180903 compared with FL180906 because we considered that the common DEGs were not the causes of different symptoms since both the isolates FL180903 and FL180906 cause ABR on fruit. Second, only DEGs showing more than 8-fold differences, the *p* value< 0.01, and the adjusted *p*-value < 0.01 were kept for further functional analysis. A total of 1124 DEGs were selected, which were considered as the DEGs of GLS- and ABR-isolate (Table S9). Looking through the DEGs, secondary metabolites, such as cytotoxin, mycotoxin, and antibiotics, were various between GC20190701 GLS- and ABR-isolate while extracellular enzymes were mostly up-regulated.

Killing the host tissues using the produced toxins, such as sesquiterpenoids is the main strategy for the full pathogenicity of necrotrophic fungal pathogens [28]. Several putative sesquiterpene synthases (STS) were revealed during plant infection in *C. graminicola* and *C. higginsianum* [29]. Cytochrome P450s (CYP) play essential roles in the fungal biosynthesis of secondary metabolites and detoxification of toxic compounds [28, 30, 31]. In this study, a total of 17 Cytochrome P450s (CYP) were revealed in the DEGs, 11 of which were up-regulated while six were down-regulated, which demonstrated that the secondary metabolites might be diversiform between GLS- and ABR-isolate. The functional roles of each CYP in the GLS- and ABR-isolate should be further explored. The methyltransferases catalyze an important pathway in the metabolism of many drugs and toxic compounds [32]. In the maize pathogen *Cochliobolus heterostrophus*, the Lae1-like methyltransferases act as a regulator of T-toxin production and thus impacts virulence to the host [33]. Five Methyltransferase proteins were revealed from the 1124 DEGs, which were all up-regulated. Those methyltransferases may involve in the pathways that increased the toxin level in GLS-isolate and therefore result in large necrotic spots and premature defoliation on leaves.

As hemibiotrophic fungi, CGSC infection procedures include penetration, growth inside living host cells (biotrophy), and tissue destruction (necrotrophy) [29]. To complete the pre-penetration, an invasion structure having mechanical pressure and some cuticle- and cell wall degrading enzymes, such as cutinases, pectinases, hemicellulases and cellulases are necessary [34]. Besides that, many enzymes, such as amylases, lipases, and proteases are also secreted to degrade plasma-membrane components and provide nutrients to help the fungus spread in plant tissue [35]. We retrieved four genes associated with the degradation of the host cell wall, which was up-regulated in GLS-isolate compared with ABR-isolate. Three genes were involved in the degradation of cellulose, and one gene was involved in the degradation of xylan (Table 1). Those results demonstrated that GLS-isolate secreted more cuticle- and cell wall degrading enzyme than that ABR-isolate, which might be the reason that GLS-isolate could infect Gala leaves and develop fast but ABR-isolate could not. However, Velho et al. proved that ABR-isolate had higher activity of pectin lyase (PNL), polygalacturonase (PG) and laccase (LAC) than GLS-isolate in culture broth [26]; nonetheless, they could not find a significant difference between the two isolates for those enzymes in infected apple leaves [26].

CGSC penetrates host epidermal cells through the melanized appressoria by producing a penetration peg, and melanization of appressoria is crucial for appressorial function [36, 37]. In the current study, we identified genes involved in melanin synthesis, such as tyrosinase and glucosyltransferase, which were highly up-regulated (Table 2). The melanin accumulation level might be higher in GLS-isolate than that in ABR-isolate causing a greater invasive ability on host plant tissues. In addition, we revealed several genes associated with pathogenicities, such as two CFEM domain-containing proteins and three carboxypeptidases that were upregulated. Combined with the above founding, we concluded that the penetration ability, pathogenicity of GLS-isolate was greater than that of ABR-isolate.

## Conclusions

The transcriptome profile between GLS- and ABR-isolate were relatively large differences, and genes involved in the secondary metabolism and extracellular enzymes were divergent. More and higher secondary metabolites were produced in GLS-isolate, the secretion of extracellular enzymes and melanin accumulation were increased, and the genes associated with pathogenicity were also up-regulated. Therefore, the pathogenicity of GLS-isolate was higher than that of ABR-isolate, which might indicate that GLS-isolate originated from ABR-isolates by mutation into a more virulent strain. The consistent deduction was concluded in previous studies [27], and the mutation was caused mainly by the high production of susceptible apple Gala acting as a selection force [2].

## Material and methods

### Fungi isolates

GLS-isolate GC20190701 and ABR-isolates FL180903, FL180906 were originated from leaves and fruits with GLS- and ABR symptoms, respectively. The GLS leaves and ABR fruit were collected from Gala and Fuji at the experimental station of Qingdao Agricultural University, Jiaozhou City of China, respectively. The causal agents were isolated from diseased leaves or fruits by a single spore isolation method as described previously [38]. The fungus was grown on potato dextrose agar (PDA) medium at 25 °C in an incubator (MGC-400HPY; Shanghai Bluepard Instruments Co. Ltd., Shanghai, China).

### Pathogenicity test assays

Conidia of the three isolates GC20190701, FL180903, FL180906 were inoculated to apple leaves and fruits to test the pathogenicity. The three isolates were incubated on a PDA medium for 3 days at 25 °C, subsequently, the mycelium was scraped from the surface of the PDA medium to induce the production of conidia. The produced conidia by each isolate were scoured into the water and adjusted to 1 × 10^4^ per mL using a hemocytometer. Twenty microliter conidia were dropped to Gala leaves or fruit by a micropipettor, and the inoculated leaves or fruit were sealed in a container, which keep moisture using a wetted tissue. The sealed containers with inoculated leaves and fruits were incubated at 25 °C in an incubator, and the symptoms caused by the three isolates were examined at 10 dpi. The pathogenicity test experiment was carried out three times at different times, and five apple leaves and five fruits were used for each treatment.

### Multi-locus phylogenetic analysis of GLS- and ABR-isolates

Multi-locus sequences were concatenated for the phylogenetic analysis. Five genes were included, such as the ribosomal internal transcribed spacer (*ITS*), actin (*ACT*), glyceraldehyde-3-phosphate dehydrogenase (*GAPDH*), chitin synthase (*CHS-1*), and β-tubulin 2 (*TUB2*). The primer pairs for amplifying target genes were ITS1F/ITS4, ACT512F/ACT783R, GDF1/ GDR1, CHS-1-79F/CHS-1-354R, and Bt2a/Bt2b, which were adopted from previous reports [39]. Multiple sequence alignments of each gene were made with the Lasergene Suite 7.1.0 software package (DNASTAR Inc. Madison, WI) [40], and manually adjusted where necessary. Aligned nucleotide sequences were used to construct phylogenetic trees using the MEGA 7.0 software (Kumar et al. 2016). The five genes’ sequences of three tested isolates have been deposited in NCBI (accessions OM818497–OM818499 for ITS, OM860286–OM860288 for ACT, OM860289–OM860291 for GAPDH, M860292–M860294 for CHS-1, and M860295– M860297 for TUB2). A phylogenetic tree was constructed based on the neighbor-joining (NJ) method and the Kimura 2-parameter method. Bootstrap resampling (1000 replications) was used to measure the reliability of individual nodes in each phylogenetic tree.

### RNA extraction, library construction, and sequencing

The three isolates GC20190701, FL180903, FL180906 were conducted transcriptome analysis. A total of nine isolate cultures, three replicas for each isolate, were subjected to RNA extraction. The isolates were incubated on a PDA medium for 96 h at 25 °C in an incubator, and mycelium was harvested from the surface of the medium for RNA extraction. Total RNA of the nine samples was extracted using the Trizol Reagent Kit (Sangon Biotech, Shanghai, China) and treated with RNase-free DNase I (TaKaRa) following the manufacturer’s protocols. Promega PolyATtract mRNA Isolation Systems were used to purified poly(A) messenger RNA (mRNA) from the total RNAs, biotinylated beads with oligo (dT) were used to enrich mRNAs. The mRNAs were fragmented into short fragments of about 300 bp in length using Magnesium RNA Fragmentation Module (New England BioLabs). Subsequently, using the short fragments as templates, the first-strand cDNA was synthesized applying random hexamer primers, and then the second-strand cDNA was synthesized by adding the buffer, dNTPs, RNase H, and DNA polymerase I. Next, the short fragments were connected with sequencing adapters with respect to the result of agarose gel electrophoresis, and suitable fragments of about 450 bp in length were selected as templates for amplification with PCR to enrich the cDNA fragments. Next, the PCR products were purified with a QIAquick PCR Purification Kit and elution in EB buffer, the obtained products were considered as the final cDNA library for sequencing. Finally, the Quality of cDNA libraries was analyzed by Agilent 2100 Bioanalyzer, and the libraries were conducted paired-end sequencing using next-generation sequencing (NGS) on Illumina HiSeq™ 4000 (Illumina, CA, USA). Preparation and sequencing of the cDNA library were implemented by Shanghai Personalbio Technology Co.,Ltd. (Shanghai, China).

### De novo assembly of sequencing reads

To cleaned-up, the raw sequencing reads, the following criteria were applied to remove the low-quality sequences: adapter and sequence less than 50 bp, and low-quality reads with more than 50% of bases with quality lower than Q20 level. The high-quality clean reads were recovered after filtering and used for transcriptome de novo assembly with Trinity software (v2.8.4). The Trinity software first combined reads with a 30 bp length of the overlap to form longer fragments without N, and these N-free assembled reads were assembled to generated transcripts. Subsequently, the generated transcripts were clustered and the longest one of each transcript was considered as a unigene. Reads counts of each unigene were calculated by mapping the transcripts back to unigenes.

### Functional annotation and differentially expressed genes

To functional analyze the transcriptomes, the assembled unigenes were firstly aligned by BLASTX to protein databases, such as NR (NCBI non-redundant protein sequences), GO (Gene Ontology), KEGG (Kyoto Encyclopedia of Genes and Genome), eggNOG (evolutionary genealogy of genes: Non-supervised Orthologous Groups), Swiss-Prot, and Pfam, to retrieve proteins with the highest sequence similarity with the unigenes along with their protein functional annotations. Then, the gene expression level was calculated with RSEM (v1.1.12) using the transcripts as a reference sequence database. The clean reads of each sample were aligned to the transcripts database, the reads of each transcript were calculated for each sample. To compare the difference of gene expression among different samples, the FPKM (Fragments per kilobase of transcript per million mapped reads) method was used for normalization [41].

The DEGs among the three isolates GC20190701, FL180903, FL180906 were identified by RSEM (RNA-Seq by Expectation-Maximization) software by the following filter criteria: |log2FoldChange| > 1, and *p*-value ≤0.05 [42].

### Gene ontology and KEGG pathway enrichment analysis

To examine the biological functions and pathways of the identified DEGs, we firstly annotated the DEGs with GO database (http://www.geneontology.org/) using a hypergeometric test [42]. GO terms that are significantly enriched in DEGs compared to the genome background were retrieved by GO functional enrichment analysis. Briefly, the GO functional enrichment analysis firstly maps all DEGs to GO terms in the database, calculating gene numbers for every term, then using the ultra-geometric test to find significantly enriched GO terms (*P*-value < 0.05) in DEGs compared to the genome background.

The significantly enriched metabolic pathways and signal transduction pathways in DEGs compared with the whole genome background identified by KEGG pathway enrichment analysis [43]. Using FDR = 0.05 and *P*-value < 0.05 as the threshold, pathways were defined as those with significant enrichment for DEGs.

### Quantitative RT-PCR validation

Total RNA was extracted from the three fungi isolates GC20190701, FL180903, FL180906 as described for the transcriptome library preparation. The purity and concentration of each RNA sample were measured in triplicate using a nanophotometer (Implen GmbH, Germany). cDNA was synthesized using the HiScript III RT SuperMix for qPCR with gDNA wiper (R323–01, Vazyme, China) according to the manufacturer’s instructions, and alpha-tubulin (TUB) gene was used as an internal control [44]. The primers for qPCR were designed according to sequences of the chosen unigenes using the PrimerQuest Tool (https://sg.idtdna.com/PrimerQuest/Home/Index). Primer sequences and unigenes are summarized in Table S10.

Quantitative PCR (qPCR) was carried out in a LightCycler® 96 System Real-Time PCR System (Roche, USA) using Taq Pro Universal SYBR qPCR Master Mix (Q712–02, Vazyme, China). The thermal cycles were set as follows: 95 °C for 30 s, followed by 40 cycles of 95 °C for 15 s and 60 °C for 30 s. The PCR products were subjected to melt curve analysis to verify the specificity. Three biological replicates and three technical replicates for each biological replicate were performed for each unigene. The *Ct* value for each biological replicate was calculated as the average value of three technical replicates. The relative expression level of each gene was calculated using the 2^−(ΔΔCt)^ method [45]. Experiments were conducted in triplicates.

## Supplementary Information

**Additional file 1: Table S1.** Summary of RNA-Seq data of the *Colletotrichum aenigma* isolate GC20190701 and *C. gloeosporioides* isolates FL180903, FL180906. Nine biological samples, three biological repeats for each isolate were sequenced using next generation sequencing on illumina sequence platform. **Table S2.** Gene annotation results of the sequenced isolates. **Table S10.** Primers used for real-time PCR to confirm RNA-Seq data.

**Additional file 2: Table S3.** Distribution of GO terms assigned to unigenes of the *Colletotrichum aenigma* isolate GC20190701 and *C. gloeosporioides* isolates FL180903, FL180906.

**Additional file 3: Table S4.** The KEGG pathway of unigenes of the *Colletotrichum aenigma* isolate GC20190701 and *C. gloeosporioides* isolates FL180903, FL180906.

**Additional file 4: Table S5.** Gene ontology enrichment of the DEGs of the *Colletotrichum aenigma* isolate GC20190701 and *C. gloeosporioides* isolate FL180906.

**Additional file 5: Table S6.** Gene ontology enrichment of the DEGs of the Colletotrichum *C. gloeosporioides isolates* FL180903 and FL180906.

**Additional file 6: Table S7.** KEGG enrichment pathways DEGs between the *Colletotrichum aenigma* isolate GC20190701 and *C. gloeosporioides* isolate FL180906.

**Additional file 7: Table S8.** KEGG enrichment pathways DEGs between the *Colletotrichum gloeosporioides* isolates FL180903 and FL180906.

**Additional file 8: Table S9.** The highly differential expression genes of GLS- and ABR-pathogen.

## Data Availability

The datasets used during the current study are available from the corresponding author on reasonable request.
