# Supplementary material for: Comparative transcriptome analysis reveals significant differences in gene expression between pathogens of apple Glomerella leaf spot and apple bitter rot
Source: BMC Genomics. 2022 Mar 31;23:246. doi: 10.1186/s12864-022-08493-w (PMC8969349; doi:10.1186/s12864-022-08493-w)
Supplement: Supplementary file 1 — Additional file 1: Table S1. Summary of RNA-Seq data of the Colletotrichum aenigma isolate GC20190701 and C. gloeosporioides isolates FL180903, FL180906. Nine biological samples, three biological repeats for each isolate were sequenced using next generation sequencing on illumina sequence platform. Table S2. Gene annotation results of the sequenced isolates. Table S10. Primers used for real-time PCR to confirm RNA-Seq data. [file 12864_2022_8493_MOESM1_ESM.docx]

**Comparative transcriptome analysis reveals significant differences in gene expression between pathogens of apple glomerella leaf spot and apple bitter rot**

Bowen Jiang, Ting Cai, Xiaoying Yang, Yuya Dai, Kaixuan Yu, Pingping Zhang, Pingliang Li, Caixia Wang, Na Liu, Baohua Li, and Sen Lian

| **Table S1 Summary of RNA-Seq data of the *Colletotrichum aenigma* isolate GC20190701 and *C. gloeosporioides* isolates FL180903, FL180906.** Nine biological samples, three biological repeats for each isolate were sequenced using next generation sequencing on illumina sequence platform. | | | | | | | | | |
| --- | --- | --- | --- | --- | --- | --- | --- | --- | --- |
| **Index** | **GC20190701_1** | **GC20190701_2** | **GC20190701_3** | **FL180903_1** | **FL180903_2** | **FL180903_3** | **FL180906_1** | **FL180906_2** | **FL180906_3** |
| No. of reads | 43,757,486 | 43,365,336 | 42,460,470 | 47,482,588 | 46,200,854 | 46,863,640 | 42,649,476 | 43,015,964 | 42,456,074 |
| No. of clean reads | 41,315,944 (94.42%) | 40,947,252 (94.42%) | 40,139,504 (94.53%) | 44,999,210 (94.76%) | 43,841,626 (94.89%) | 44,424,662 (94.79%) | 40,489,708 (94.93%) | 40,745,532 (94.72%) | 40,304,008 (94.93%) |
| Bases (bp) | 6,563,622,900 | 6,504,800,400 | 6,369,070,500 | 7,122,388,200 | 6,930,128,100 | 7,029,546,000 | 6,397,421,400 | 6,452,394,600 | 6,368,411,100 |
| Clean Data (bp) | 6,197,391,600 (94.42%) | 6,142,087,800 (94.42%) | 6,020,925,600 (94.53%) | 6,749,881,500 (94.76%) | 6,576,243,900 (94.89%) | 6,663,699,300 (94.79%) | 6,073,456,200 (94.93%) | 6,111,829,800 (94.72%) | 6,045,601,200 (94.93%) |

| **Table S2 Gene annotation results of the sequenced isolates** | | |
| --- | --- | --- |
| **Database** | **Number** | **Percentage (%)** |
| NR | 23,089 | 59.44 |
| GO | 14,445 | 37.19 |
| KEGG | 7,662 | 19.73 |
| Pfam | 10,049 | 25.87 |
| eggNOG | 19,696 | 50.71 |
| Swissprot | 15,372 | 39.57 |
| In all database | 3,655 | 9.41 |

| **Table S10 Primers used for real-time PCR to confirm RNA-Seq data** | | | | | |
| --- | --- | --- | --- | --- | --- |
| **Gene ID** | **log2^FoldChange^** | **Gene Length (bp)** | Primers | **Primer sequences** | **Product length (bp)** |
| DN21088_c0_g1 | 3.7560 | 1791 | 20188-F | GCGTCGAATGGCTCATAGAA | 129 |
|  |  |  | 20188-R | GAGAGTCGGGCATACCATTATC |  |
| DN26894_c0_g1 | 4.4476 | 1644 | 26894-F | GAGACTCGGTGCAGCTAAAT | 85 |
|  |  |  | 26894_R | CGTCGCCGTTGTTCAGATA |  |
| DN30086_c0_g1 | 3.3697 | 1749 | 30086-F | CAACACCAGGCCAGACTAAA | 110 |
|  |  |  | 30086-R | AAGCTGGTCACCTGTTGAAG |  |
| DN28222_c0_g1 | -5.0447 | 2290 | 28222-F | GAGACGCCGAGCTTATCAAA | 105 |
|  |  |  | 28222-R | GACGATTGCCGTAGTCTTTCT |  |
| DN28743_c0_g2 | -8.5906 | 1287 | 28743-F | TGAGCGCGTAGTATCTCTCT | 102 |
|  |  |  | 28743-R | TCACCTCGACCTTTGTTGAC |  |
| DN36415_c8_g1 | -12.2141 | 791 | 36451-F | CTGAGTGTCGAGATCGAAGAAG | 101 |
|  |  |  | 36451-R | CTACTTACATGCGTCCTCCTTAC |  |
| Alpha-tubulin | - | - | Tub-a-F | CCACTTCCCTTTGGTCGCTTAC | 85 |
|  | - | - | Tub-a-R | CATGGTCATCTCCTGGACAGAGT |  |
